# Complete Genome Sequence of *Curtobacterium* sp. Strain YC1, Isolated from the Surface of Nostoc flagelliforme Colonies in Yinchuan, Ningxia, China

**DOI:** 10.1128/MRA.01467-20

**Published:** 2021-03-11

**Authors:** Yongjun Wei, Xiang Gao

**Affiliations:** a Key Laboratory of Advanced Drug Preparation Technologies, Ministry of Education, School of Pharmaceutical Sciences, Zhengzhou University, Zhengzhou, Henan Province, China; b School of Food and Biological Engineering, Shaanxi University of Science and Technology, Xi’an, Shaanxi Province, China; University of Arizona

## Abstract

The genus *Curtobacterium* belongs to the family *Microbacteriaceae*, within the phylum *Actinobacteria.* This genus includes a wide range of Gram-positive species associated with plants and soils. Here, we report the genome sequence of a new strain, *Curtobacterium* sp. strain YC1, which was isolated from the surface of Nostoc flagelliforme colonies. The genome of this strain contains one chromosome and one plasmid, and its size is 3.4 Mb.

## ANNOUNCEMENT

Nostoc flagelliforme is a type of terrestrial filamentous cyanobacterium distributed in the arid and semiarid steppes of the western and northwestern parts of China (1). It appears as a hair-like colony on the soil surface. It has been used as a healthy food for more than 2,000 years, and disordered exploitation during the past century has caused a sharp decline in its availability. The State Council of China banned the collection and trade of N. flagelliforme in 2000.

A previous study suggested that *Curtobacterium* species could generate oxidative stress to inhibit plant growth (2). We thus attempted to isolate and identify those epiphytic microorganisms on the surface of N. flagelliforme colonies collected on the eastern side of Helan Mountain in Yinchuan, Ningxia, China (3). The epiphytic microorganisms were washed from the N. flagelliforme colonies with sterilized water. The collected water was plated onto Luria-Bertani (LB) solid medium supplemented with 5-bromo-4-chloro-3-indolyl-β-d-galactopyranoside (X-Gal) (used to detect β-galactosidase activity), and one strain that showed β-galactosidase activity was isolated. The strain was identified as a new *Curtobacterium* strain by 16S rRNA gene sequencing and was named *Curtobacterium* sp. strain YC1.

One done of the isolated *Curtobacterium* sp. strain YC1 was cultivated in LB medium and collected for DNA extraction using the DNeasy PowerMax soil kit (Qiagen). The Illumina HiSeq 4000 platform was used to obtain the draft genome assembly of *Curtobacterium* sp. YC1, and the PacBio RS II platform was used to help obtain the full genome assembly. The same DNA was used for Illumina and PacBio sequencing at the Beijing Genomics Institute (BGI) (Shenzhen, China). Paired-end sequencing (2 × 150 bp) was used for Illumina sequencing, and the total read number was 8,353,300. The total Illumina data represented 1,148 Mbp, and the coverage was 339×. For PacBio sequencing, a total of 91,229 reads were obtained, and the read *N*_50_ was 19,715 bp. The total PacBio data represented 1,280 Mbp, and the coverage was 378×. The program pbdagcon (https://github.com/PacificBiosciences/pbdagcon) was used for self-correction of the PacBio sequencing results (4), and draft genome unitigs were assembled with Celera Assembler v8.3 against a high-quality corrected circular consensus sequence subread set (5). GATK v1.6-13 and the SOAP tool package (SOAP2, SOAPsnp, and SOAPindel) were used to improve the accuracy of the genome sequences by making single-base corrections (6, 7). The bacterial plasmid database was used to identify plasmid sequences in the genome using SOAP2 software. Genes in the genome were predicted by Glimmer3 (http://ccb.jhu.edu/software/glimmer/index.shtml) with hidden Markov models. The KEGG, Clusters of Orthologous Groups of proteins (COG), nonredundant, Swiss-Prot, and Gene Ontology (GO) databases were used for functional gene annotation (8). Noncoding RNA sequences were recognized with the tools tRNAscan-Se v1.3.1, RNAmmer v1.2, and the Rfam database v9.1 (9–11). Default parameters were used for all software unless otherwise specified.

**FIG 1:**
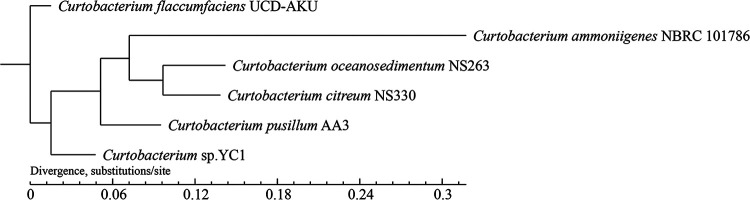
Core/pan-gene-based phylogenetic tree of *Curtobacterium* sp. strain YC1 and its closest relatives. MUMmer v3.22 and BLASTp were used to calculate the synteny of five selected *Curtobacterium* species and *Curtobacterium* sp. strain YC1. The core/pan genes of the six *Curtobacterium* species were clustered by the CD-HIT program for rapid clustering of similar proteins with a threshold of 50% pairwise identity and 0.7 length difference cutoff in amino acids. The gene family was built from the genes of the six *Curtobacterium* species with several software tools. Finally, the phylogenetic tree was constructed by TreeBeST v1.9.2 using the neighbor-joining method based on the gene family clustering with Muscle software v3.8.31. The results suggested that *Curtobacterium* sp. YC1 is a new *Curtobacterium* strain.

One circular chromosome assembly and one circular plasmid assembly composed the final assembly of the *Curtobacterium* sp. YC1 genome. The length of the chromosome is 3,301,309 bp, with a GC content of 71.68%. The length of the plasmid is 77,217 bp, with a GC content of 66.99%. There are 3,270 genes in the chromosome and the plasmid, and the average gene length is 938 bp. The length of all the genes represented 90.79% of the whole genome. Among the 3,270 genes, the Swiss-Prot database identified 1,119 genes, the COG database identified 2,315 genes, the nonredundant database identified 3,083 genes, the KEGG database identified 1,683 genes, and the GO database identified 1,918 genes. A total of 84 genes were identified with the carbohydrate-active enzyme (CAZy) database, and 52 glycoside hydrolase genes were annotated (12). There are 46 copies of tRNAs, 3 copies of 5S rRNAs, 3 copies of 16S rRNAs, 3 copies of 23S rRNAs, and 2 copies of small RNAs. Moreover, 6 clustered regularly interspaced short palindromic repeat (CRISPR) regions were predicted with CRISPRFinder (https://crispr.i2bc.paris-saclay.fr/). The phylogenetic analyses suggested that *Curtobacterium* sp. YC1 is a new *Curtobacterium* strain (Fig. 1). The public version of this genome was annotated using PGAP through NCBI (13).

### Data availability.

The complete genome sequences (one chromosome and one plasmid) of *Curtobacterium* sp. YC1 were deposited in GenBank under the accession numbers CP066341 and CP066342, with BioProject accession number PRJNA683987, BioSample accession number SAMN17050104, and SRA accession numbers SRR13298322 and SRR13638075.
